# Introducing Micrometer-Sized Artificial Objects into Live Cells: A Method for Cell–Giant Unilamellar Vesicle Electrofusion

**DOI:** 10.1371/journal.pone.0106853

**Published:** 2014-09-17

**Authors:** Akira C. Saito, Toshihiko Ogura, Kei Fujiwara, Satoshi Murata, Shin-ichiro M. Nomura

**Affiliations:** 1 Department of Bioengineering and Robotics, Tohoku University, Aoba-ku, Sendai, Japan; 2 Department of Developmental Neurobiology, Institute of Development, Aging and Cancer (IDAC), Tohoku University, Aoba-ku, Sendai, Japan; 3 CREST, JST, Tohoku University, Sendai, Miyagi, Japan; 4 Department of Biosciences and Informatics, Keio University, Hiyoshi, Kohokuku, Yokohama, Japan; 5 JSPS Research Fellow, Japan Society for the Promotion of Science, Chiyoda-ku, Tokyo, Japan; Texas A&M University, United States of America

## Abstract

Here, we report a method for introducing large objects of up to a micrometer in diameter into cultured mammalian cells by electrofusion of giant unilamellar vesicles. We prepared GUVs containing various artificial objects using a water-in-oil (w/o) emulsion centrifugation method. GUVs and dispersed HeLa cells were exposed to an alternating current (AC) field to induce a linear cell–GUV alignment, and then a direct current (DC) pulse was applied to facilitate transient electrofusion. With uniformly sized fluorescent beads as size indexes, we successfully and efficiently introduced beads of 1 µm in diameter into living cells along with a plasmid mammalian expression vector. Our electrofusion did not affect cell viability. After the electrofusion, cells proliferated normally until confluence was reached, and the introduced fluorescent beads were inherited during cell division. Analysis by both confocal microscopy and flow cytometry supported these findings. As an alternative approach, we also introduced a designed nanostructure (DNA origami) into live cells. The results we report here represent a milestone for designing artificial symbiosis of functionally active objects (such as micro-machines) in living cells. Moreover, our technique can be used for drug delivery, tissue engineering, and cell manipulation.

## Introduction

Direct introduction of functional objects into living cells is a major topic in biology, medicine, and engineering studies, since such techniques facilitate manipulation of cells and allows one to change their functional properties arbitrarily. In order to introduce various objects into cells, several methods have been developed, for example, endocytosis and macropinocytosis [1]–[3]. Nonetheless, the sizes of introducible objects are largely limited: up to several hundred nanometers and a few micrometers in diameter. In addition, the uptake of objects is dependent on cell type, and neither endocytosis nor macropinocytosis occur, for example, in lymphocytes. Even after successful endocytosis, incorporated objects are transported to the endosomes; they are then eventually transferred to the lysosome, in which acidic hydrolases degrade the materials. Hence, these two systems are not particularly suitable for introduction of functionally active molecules and objects. To overcome these obstacles, novel delivery systems have been contrived, such as cationic liposomes and nanomicelles, that are used for gene transfer; yet, only nucleic acids that are limited to a few hundred nanometers in size can be introduced [4]–[8]. By employing peptide vectors, comparatively larger materials can be introduced into cells, although the size limit of peptides and beads is approximately 50 nm [9], which is again insufficient for delivery of objects, such as DNA origami [10], [11] and larger functional beads.

On the other hand, several methods have been established that allow penetration of the cell membrane; these include microinjection, electroporation, and electrofusion. Cell–cell electrofusion is a traditional technology for generating hybridomas and involves fusing adjoining cell membranes. Based on this classical technique, Shirakashi *et al.* proposed cell and giant unilamellar vesicle (GUV) fusion, by which GUVs loaded with low-molecular weight oligosaccharides were fused with Jurkat cells [12]. Nonetheless, the efficiency of transfer of the GUV contents (trehalose, raffinose, and KCL in this case) and cell viability were not measured in that study, and only cell–GUV electrofusion geometry was evaluated microscopically and confirmed theoretically by a Finite-Element-Method analysis of the electric field around the fused cells. The GUVs were prepared by a conventional electroformation technique, which is quite a limited method for enclosing large-sized objects into GUVs.

To date, standard methods have been used for preparation of giant vesicles [13], [14], that encapsulate substances; however, few reports have described the encapsulation of micrometer-sized substances into giant vesicles at high volume fractions [15]. Recently, the water-in-oil (w/o) emulsion centrifugation method has been developed. With this new technique, it is now possible to prepare GUVs that contain artificial materials larger than 1 µm in diameter [13], [15]–[18].

Here we adopt the w/o emulsion centrifugation method to entrap various large artificial objects (up to 1 µm in diameter) in GUVs. After cell–GUV electrofusion, the objects were transferred into live cells, which retained high viability, and, more importantly, underwent several rounds of normal cell division. Based upon these observations, this method can be used in various experimental situations, namely, simultaneous transfer of multiple genes, proteins, and small molecules for generation of induced pluripotent stem (iPS) cells, and even for creation of artificial cells that bear molecular robots (e.g., DNA nanostructures and DNA devices) in the cytosol.

## Materials and Methods

### Artificial objects for transfer

In general, negatively charged materials do not adhere well to cell surfaces. To avoid non-specific absorption to the cell surface, we used negatively charged lipids and materials for this experiment; i.e., dioleoylphosphatidylglycerol (DOPG), carboxylated beads, plasmid DNA, and DNA origami. Fluorescent microbeads (FluoSpheres, carboxylate modified; 0.2, 0.5, 1.0, and 2.0 µm in diameter, 2 mM surface azide group; λ_Ex_/λ_Ex_ = 505/515 nm) were purchased from Invitrogen. The initial bead concentration for forming GUVs was 40 µM. An EGFP and mCherry expression vector (pEGFP-C1, pmCherry) were prepared using a NucleoBond Xtra Midi plus kit (Macherey-Nagel GmbH & Co., Düren, Germany), according to the manufacturer's instructions. The calculated concentration of the EGFP and mCherry plasmid entrapped in GUVs was 220 and 230 ng/µl, respectively.

DNA origami with a chipped rectangular shape (60×90 nm; Figure S1 in File S1) was designed using caDNAno software (http://cadnano.org). Table S1 in File S1 shows the complete sequence of the DNA origami. The assembly of the structure was checked by electrophoresis and atomic force microscopy (Figure S2 in File S1). DNA origami was loaded into GUVs at a final concentration of 3.36 nM.

### GUV preparation by the w/o emulsion centrifugation method

GUVs were prepared using the water-in-oil (w/o) emulsion centrifugation method, with modifications [16]–[18]. Dioleoylphosphatidylcholine (DOPC, NOF, Japan), DOPG (NOF, Japan), and cholesterol (Wako, Japan), at a weight ratio of 18∶2∶1 (total: 105 mg), were dissolved in 1050 µl chloroform. This solution was poured into a glass tube (10 mm ø), then first dried under argon gas and subsequently under vacuum, and was then mixed with 500 µl of liquid paraffin (Wako, Japan). The mixture was treated by ultrasonication at 60°C for 60 min. Artificial objects (fluorescent microbeads, DNA origami, or plasmid DNA) were mixed with the inner solution (consisting of 90 mM sucrose, 210 mM mannitol, 0.1 mM CaCl_2_, 0.1 mM MgCl_2_, and target solution), and 50 µl of the inner solution was then added to the lipid mixture. Then, the tube was vortexed for 1 min to create a micrometer-sized W/O emulsion. The emulsion was poured gently onto the outer solution (consisting of 300 mM mannitol, 0.1 mM CaCl_2_, 0.1 mM MgCl_2_). After centrifugation at 18,000×*g* for 30 min at 4°C, the emulsion was passed through the w/o interface saturated with lipids to form a bilayer membrane. To avoid mixing between oil and water, GUVs were extracted from the bottom of the tube through a hole made using a syringe needle (G25, Terumo, Japan). The average diameter of the GUVs was calculated from microscopic images to be 37±13 µm (see Figure S3 in File S1). The number of beads entrapped in each GUV was calculated to be in the order of 10^1^–10^4^ from fluorescent microscopic images. To confirm that the efficiency of introduction of foreign objects is dependent on size, we prepared GUVs by entrapping several sets of microbeads (0.2, 0.5, 1, and 2 µm). The efficiency of entrapment of the beads in GUVs was estimated by flow cytometry. The values were 99.1, 91.6, 81.9, and 67.3% for beads of 0.2, 0.5, 1, and 2 µm in diameter, respectively. The microscopic images obtained immediately prior to electrofusion are shown in Figure S4 in File S1.

### Cell culture

HeLa cells (obtained from ATCC, CCL-2) were cultured in DMEM buffer (Gibco Invitrogen, Grand Island, NY, USA) supplemented with 10% fetal bovine serum (Biowest, France) and 1% antibiotic–antimycotic (Gibco Invitrogen, Grand Island, NY, USA). Cells were seeded onto plastic- or glass-based dishes (diameter: 35 mm) and maintained in a 5% CO_2_ incubator (Astek SCA-80D, Japan) at 37°C for 1, 2, 3, or 5 days.

### Cell–GUV electrofusion

**Figure 1:**
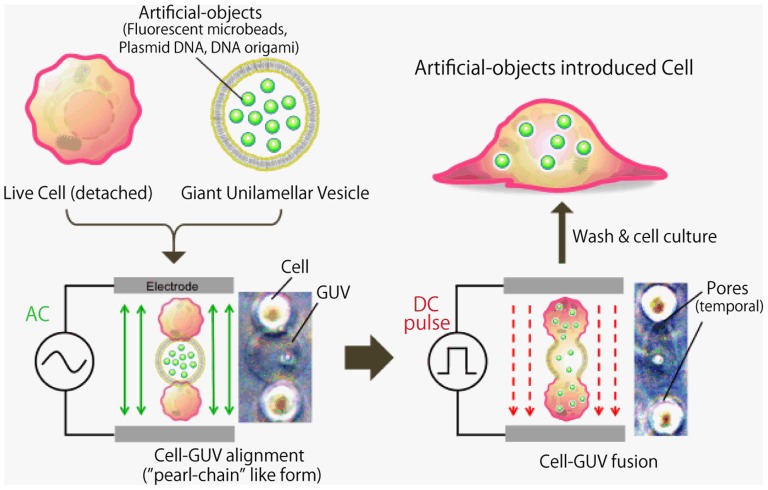
Schematic diagram of the cell–GUV electrofusion process. Cells and GUVs are exposed to an alternative current (AC) field to induce cell alignment (chain-of-pearls-like structure), and are then pulsed with direct current (DC) voltage to create breaks in the contact region between the cell membrane and GUV surfaces. Representative images are shown to demonstrate the appearance of cell–GUVs that had been exposed to the AC field and DC pulse.

A schematic diagram of the electrofusion setup is shown in Figure 1. Cultured HeLa cells (confluent) were removed by adding 0.25% trypsin–EDTA solution. The cells were washed twice with phosphate-buffered saline (PBS) and then suspended in the fusion buffer (300 mM mannitol, 0.1 mM CaCl_2_, 0.1 mM MgCl_2_). An aliquot of 400 µl of GUVs in mannitol solution and 400 µl of cell suspension were placed into the electrofusion chamber (NEPA GENE, Japan) that was connected to the electricity generator of the cell electrofusion equipment (ECFG21, NEPA GENE, Japan). The GUV concentration (2×10^4^ GUVs/ml) was estimated from microscopic images. The cell concentration used for fusion was 1×10^5^ cells/ml. The chamber consisted of two parallel platinum electroplates (2-mm thickness, 80-mm length, 5-mm height, 2-mm gap). The solution was exposed to a 15 V/mm alternating current (AC) field for 30 s to induce a cell and GUV alignment, known as a chain of pearls. Then, the solution was exposed to 175 V/mm of direct current (DC) pulse for 50 µs five times, to induce cell–GUV fusion. In this method, cell–GUV fusion is triggered by the irreversible electrical breakdown of the membranes in the contact region. Then, the cell membrane was treated with post-electrofusion 15 V/mm alternating current (AC) field for 10 s to induce maintenance of the cell and GUV contacts. Thereafter, the cells were washed three times with PBS and placed into a cell culture dishes containing DMEM.

### Flow cytometric analysis

Flow cytometric analysis was performed to measure the quantity of fluorescent microbeads introduced into the cells by the cell−GUV electrofusion method. After electrofusion, HeLa cells were seeded in 35-mm dishes and cultured for two days. The cells were removed by adding 0.25% trypsin–EDTA solution, and then counted using flow cytometer (Cell Lab Quanta SC MPL, Beckman Coulter, USA) equipped with a blue laser (488 nm). A screening gate was set on the electronic cell volume vs. SSC plot, to allow analysis only of cells of a size with a diameter in the range of 11–16 µm.

### Microscopy and image acquisition

For fluorescence observation, cells were fixed with 1% paraformaldehyde (Wako, Japan) for 30 min, washed with PBS (Gibco Invitrogen, Grand Island, NY, USA), incubated with the 10-µM cell tracker Red CMTPX (Life Technologies, USA) in DMSO for 15 min at 25°C. Thereafter, cells were washed with PBS, and then incubated with 0.7 µg/ml Hoechst 33342 (Invitrogen, USA) for 15 min. Fluorescence images and phase contrast images were acquired using a highly sensitive color camera (DP-73, Olympus, Japan) attached to an inverted fluorescent microscope (IX-71, Olympus, Japan). Cross sectional images of cells were obtained using a confocal microscope (FV-1200, Olympus, Japan) with a set of lasers (405, 473, and 543 nm).

## Results

### Cell–GUV electrofusion

**Figure 2:**
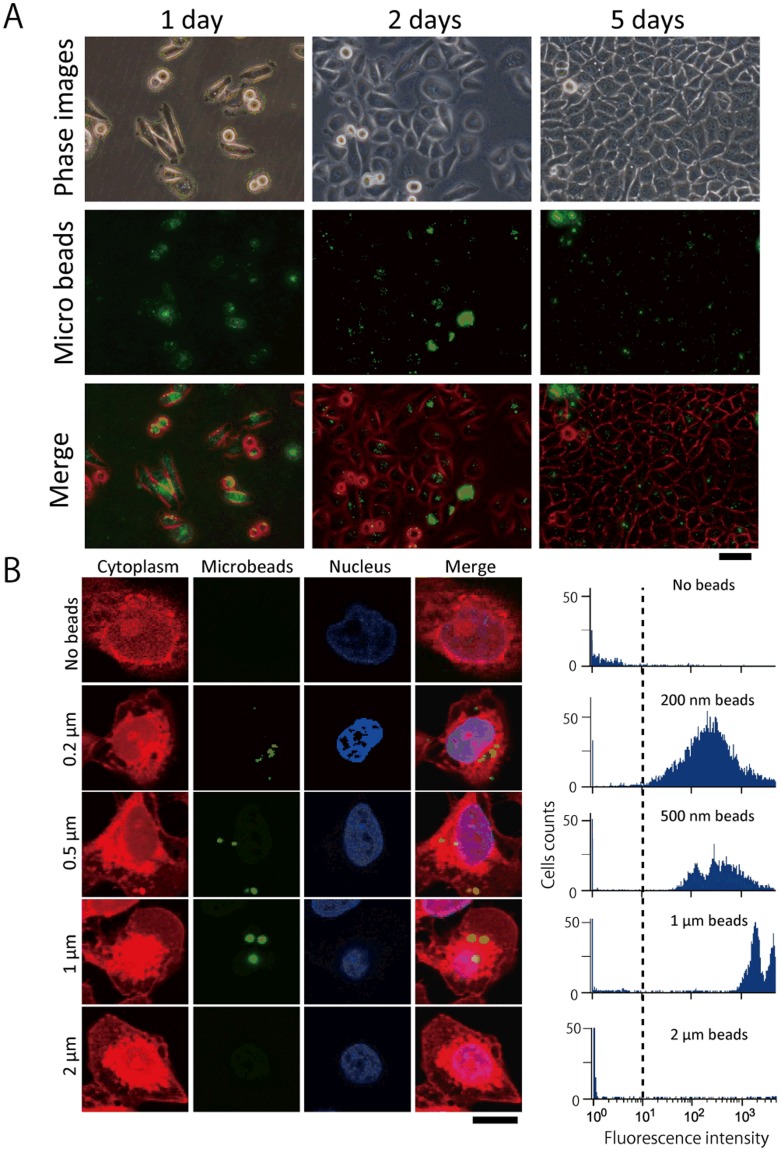
Representative images of cultured HeLa cells containing fluorescent microbeads introduced by cell–GUV electrofusion. (A) Microscopic images of cells that had been cultured for 1 (left column), 2 (middle column), or 5 (right column) days after the electrofusion process. The diameter of the beads used here was 0.2 µm. Phase contrast gray scale images were converted to red-scale. Scale bar  = 50 µm. (B) Fluorescent beads with various diameters were introduced into HeLa cells by cell–GUV electrofusion. Left: Confocal microscopic images show the cross-section of the treated HeLa cells. These images show HeLa cells into which (from the top) no beads, or beads of 0.2 µm, 0.5 µm, 1 µm, and 2 µm diameter (green) had been introduced. The cytoplasm is shown in red and nuclei in blue, and merged images are shown in the right column. Scale bar  = 10 µm. Right: Flow cytometric detection of microbeads introduced into HeLa cells. Single parameter histograms of the cell number versus log fluorescence intensity are shown. The histograms represent a total of 7,000–15,000 cells counted for each measurement.

We first introduced fluorescent microbeads into HeLa cells by electrofusion with GUVs. After application of an AC field and a DC pulse, the suspension of cells was cultured. Figure 2A shows the microscopic images of the HeLa cells with the introduced 0.2-µm microbeads. Prior to observation, these cells were thoroughly washed with PBS at 3 h after cell fusion. The treated cells survived for at least 5 days and proliferated until confluence was reached. The number of trials for each experiment was greater than five.

The treated cells were cultured for 3 days after fusion with GUVs containing the beads. Figure 2B (left) shows a series of confocal microscope images; in these images, 0.2, 0.5, and 1-µm microbeads can be observed inside the cells. However, 2-µm microbeads were not observed in the cells. The Z-stack images for each size of microbeads introduced into HeLa cells are shown in Figure S5 in File S1.

### Flow cytometric quantification of the microbeads introduced into cells

The percentage of HeLa cells containing the introduced microbeads was quantified by flow cytometry. The cells were cultured for 2 days after electrofusion. Figure 2B (right) shows histograms of the cells emitting a green fluorescent signal. The values, shown in the inset, for 0.2, 0.5, 1, and 2-µm microbeads, and no microbeads, were 73, 50, 40, 0.38, and 0.31% respectively. These values were defined as the ratio of cells that demonstrated a fluorescence intensity of more than 10. These data revealed a threshold between 1 and 2 µm for the size of beads introduced into live cells by using cell−GUV electrofusion.

### Introduction of plasmids and DNA origami into the cell

**Figure 3:**
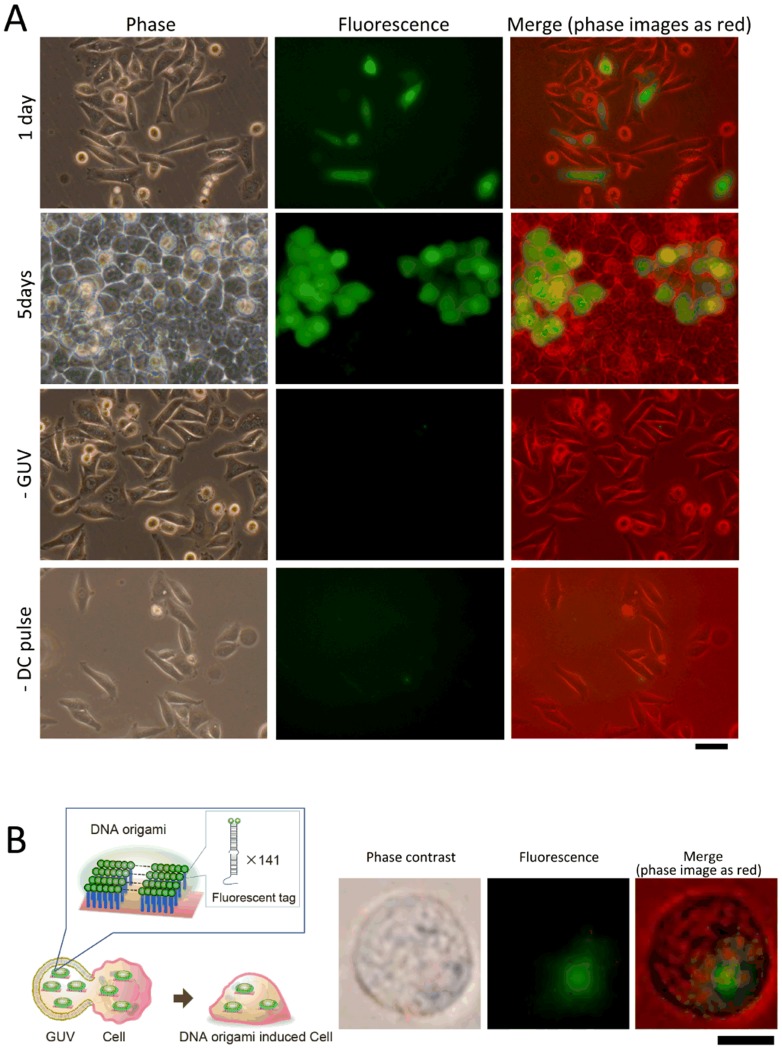
Biomaterials introduced into HeLa cells by cell–GUV electrofusion. (A) Plasmid DNA encoding EGFP (pEGFP; CMV promoter, 4.7 kbp) was adopted as a reporter. pEGFP was introduced into HeLa cells and cells were then cultured for 1 or 5 days. From the top row, images obtained by fluorescent microscopy are shown for GUV-treated cells after culturing for 1 day, 5 days, cells lacking GUVs (1 day), or not exposed to the DC pulse (1 day). Phase contrast (left column), EGFP expression in cells (green, middle column), and merged images (phase contrast shown in red-scale; right column) are shown. Scale bar  = 50 µm. (B) An artificially designed DNA nanostructure (origami) was also introduced into HeLa cells. The DNA origami structure was labeled with green fluorescent dye (by using FITC-conjugated oligonucleotides, 282 FITC molecules per single origami). Corresponding images of phase contrast (left column), DNA origami (green, middle column), and merged images (phase contrast shown in red-scale) were obtained by fluorescent microscopy immediately after cell–GUV electrofusion. Scale bar  = 10 µm.

We also investigated the introduction of an EGFP-encoding plasmid (pEGFP) and DNA origami into the cells by cell–GUV electrofusion (Figure 3). In Figure 3A, we show phase contrast and fluorescent microscopy images of HeLa cells into which pEGFP had been introduced and which had then been cultured for 1 and 5 days prior to observation. The number of EGFP-expressing cells was counted from the fluorescent microscopic images. The transfer efficiency of pEGFP was estimated to be approximately 20%. No EGFP signal was observed for cells that had not been fused with GUVs or had not been exposed to DC pulses (Figure 3A).

We also introduced fluorescence-labeled DNA origami into HeLa cells. The origami structure was designed to allocate 282 fluorescent FITC-labels onto an area of 60×90 nm^2^. The cell image in Figure 3B was obtained by fluorescence microscopy immediately after the electrofusion treatment; the green fluorescent spot indicates the position of the FITC-tagged DNA origami. When we used a bare fluorescently modified oligonucleotide, without DNA origami, no fluorescent signal was observed in the live cells. We noted that the fluorescent signal of the introduced origami disappeared after overnight culturing.

### Introduction of multiple artificial objects into the cells

**Figure 4:**
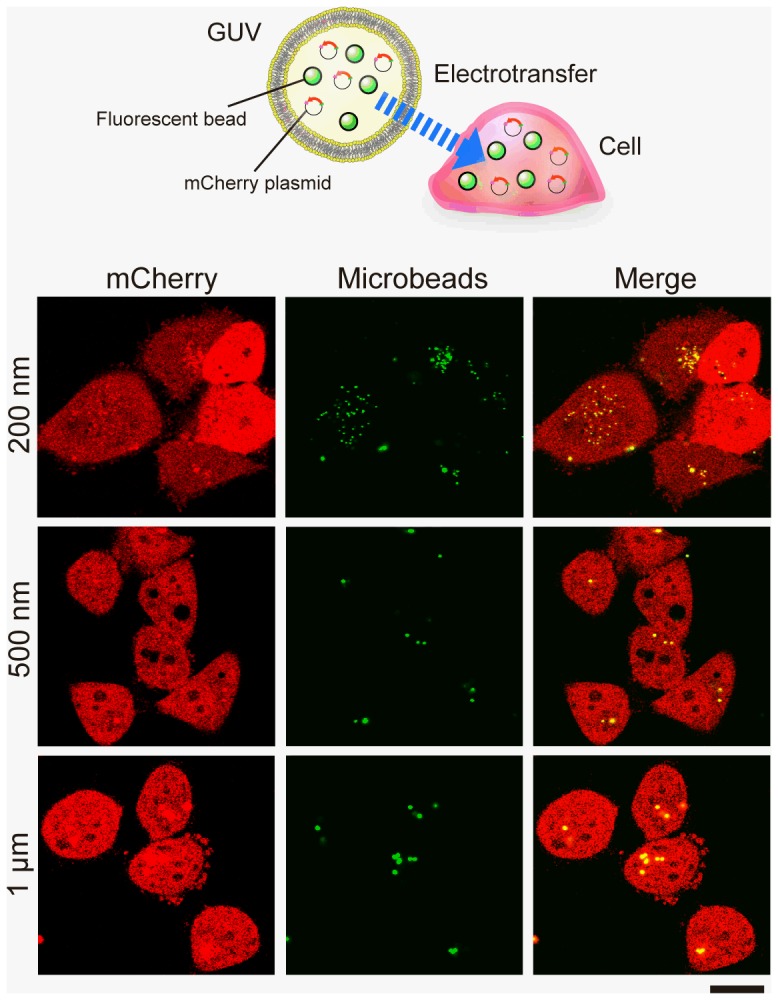
Introducing multiple components of microbeads and plasmids into live cells by cell–GUV electrofusion. GUVs including both the plasmid mCherry and fluorescent microbeads were prepared for electrofusion with HeLa cells. The treated cells were then cultured for 2 days. Confocal microscopic images show the cross-section of the treated HeLa cells into which (from the top) beads of 0.2 µm, 0.5 µm, and 1 µm diameter (green) had been introduced. The mCherry expression in cells is shown in red, and merged images are shown in the right column. Scale bar  = 20 µm.

We then confirmed whether it is possible to introduce multiple artificial objects simultaneously into the live cell using our method. We prepared GUVs entrapping both the pmCherry (red fluorescent protein-encoding plasmid) and fluorescent microbeads of different sizes (0.2, 0.5, and 1 µm in diameter). After electrofusion, the treated HeLa cells were then been cultured for 2 days prior to observation. Confocal microscopic images show the results of cell–GUV electrofusion experiments (Figure 4). In these images, 0.2, 0.5, and 1-µm microbeads were observed inside the Hela cells, which showed red fluorescence derived from the mCherry expression plasmid that was introduced into the cells along with the beads.

## Discussion

In this study, we demonstrated that the cell–GUV electrofusion method can be used to introduce artificial objects of up to at least 1-µm in size into cells, and that the hybrid cells had a viability similar to that of normal cells.

Shirakashi *et al.* proposed that application of a high-voltage DC pulse induces breakdown of the contact zones of cell and GUV membranes [12], creating a small passage between them. In their report, they concluded that an oligosaccharide solution could be transferred into the cells in this way [15], although neither the transfer efficiency nor the cell viability after electrofusion was demonstrated. Furthermore, solid objects, such as fluorescent beads and DNA origami, were not used as GUV contents, and only water-soluble substances (trehalose, raffinose, and KCL) were investigated. Hence, the possibility of using solid objects remained unanswered. Here, we showed that large solid materials can be introduced into cells safely and efficiently using our modification of this technique.

From our results, it appears that objects in the GUVs can pass through the pores formed in the contact zones of cell and GUV membranes, and then move into the cytosol. Osmotic pressures in the cells and GUVs are similar (298 vs. 300 mOsm/kg, respectively); hence, osmotic pressure would not be a force that drives the movement of the GUV contents, although balance of the pressures between cells and GUVs could prevent unpredictable cytosolic flow and maintain high cell viability.

The live cell membrane is lined with a cortex consisting of a mesh-like protein structure, called the membrane cytoskeleton [19]–[21]. Before entering into the cytosol, moving objects pass through the pore formed in the cell membrane, and then collide with the meshwork, which is an approximately 70-nm mesh cytoskeletal protein network in the case of HeLa cells [19]. Since our results showed that electrofusion can introduce plastic microbeads up to 1-µm in diameter, which then moved into the cytosol, the meshwork could be stretched and dragged open, transiently, along with expansion of the electrically formed pore.

In this study, plasmid DNA was also transferred into live cells, and these expressed green fluorescence. We could demonstrate that the plasmids were delivered into cytosol. This method does not require potentially toxic chemicals, such as the positively charged lipids that are required for gene transfection. Although further optimization of the process will be investigated, the low transfer efficiency of plasmids (approximately 20%) is attributable to the low density of both the dispersed cells and the GUVs used for fusion.

Many types of molecular devices based on DNA nanotechnology have been designed to be functional within the cellular cytosol [22]–[25]. The fates of artificial DNA nanostructures in cells remain unclear. To perform its function continuously in live cells, it seems that the introduced DNA nanostructure must be stabilized and protected from degradation by chemical modification. With our cell−GUV electrofusion technique, one can introduce a spherical object of up to 1 µm in diameter. This size allowance enables direct introduction of DNA with some protective DNA-binding compounds, such as biocompatible polycations, and even artificial chromosomal structures. In our trial, the adopted DNA origami was too large to pass through the nuclear pore (−10 nm in diameter). However, if the stability of the DNA origami in the cell is high enough to survive several rounds of cell division, it can be incorporated into the nucleus and act as an alternative source of genetic information.

Constructing a cytoskeletal mesh that lines the newly enhanced membrane region is time-consuming (requiring about 15–20 min.) [26]. Resealing the cell membrane pore caused by the 200 V/mm electric pulse requires several hundred seconds [27]. Under our experimental conditions, the membrane surface tension on the cell-side, which is reinforced by the cytoskeleton, is greater than that on the GUV-side. It is unlikely that the fused membrane can maintain this tension difference until the cytoskeleton has been reconstructed totally. In addition, the duration of pulsing and the post fusion time (about 10 s) is much less than the time needed for reconstruction of the cytoskeleton. Thus, we conclude that the “membrane fusion” in our experiment seems to be quite a limited and temporary event. After the electrical treatment, the GUVs must have been separated from the cell again during the washing stage. Although the word “fusion” does not adequately express the entire phenomenon, contents of the GUV are transferred into live cells via the electrical treatment. The detailed mechanisms underlying the GUV content transfer should be investigated from a cell scientific point of view. For now, we have termed this method “cell–GUV electrotransfer”.

Our method reported here could contribute to efficient introduction of artificial structures and materials, such as large magnetic beads, Yamanaka four factors, in either the form of DNA or protein, for the production of iPS cells, and chemically modified beads, into live cells. Moreover, in future, direct introduction of a systematic molecular device complex [28], a type of molecular robot, into the cellular cytosol should be tested. These bioengineered hybrid cells are likely to be useful for drug delivery, tissue engineering, and elucidation of cell mechanisms in future.

## Supporting Information

File S1 **Figure S1: Schematic diagram of the square DNA origami structure.** M13mp18 ssDNA, its complementary ssDNA sets (called staples), and green fluorescent (FITC)-conjugated oligonucleotides (5′-GCAATGAGTAGATCCTGGCACTCTCGATGCGACAG-3′ and 5′-TGCCAGGATCTACTCATTGC-3′) were purchased from Operon Technologies (Japan) and Takara Bio (Japan), respectively. These DNAs were mixed (M13:staples:FITC  = 4 nM:20 nM:60 nM) and annealed in a buffer (50 mM NaCl, 10 mM Tris-HCl, 10 mM MgCl_2_, 1 mM DTT, pH 7.9, 25°C) for 3.5 h across a temperature range from 95 to 25°C at a rate of -1°C/3 min. **Figure S2: Identification of the DNA origami structure.** (A) Electrophoresis analysis. Left, middle, and right lanes contain the marker, bare plate-like DNA origami structure, and fluorescently-tagged DNA origami, respectively. The samples were analyzed by 1% agarose gel electrophoresis (100V, 1 hour). The DNA origami structure, with or without fluorescent (FITC) tag, was electrophoresed in a 1% agarose gel that was exposed to DC 100 V for 1 h. FITC fluorescence was detected using a ChemiDoc MP system (BioRad, Japan). A band showing FITC-tagged origami was clearly observed under blue light. (B) AFM images for the DNA origami. The AFM image was obtained on an AFM system (Nano Live Vision, RIBM, Tsukuba, Japan) using a silicon nitride cantilever (resonant frequency  = 1.5 MHz, spring constant  = 0.1 Nm^-1^, EBDTip radius  = 24 nm, Olympus BL-AC10DS-A2). The sample (2 µL) was adsorbed onto a freshly cleaved mica plate for 5 min at room temperature, and then washed twice with the same buffer solution. Scanning was performed in the same buffer solution using a tapping mode. The final concentration of the DNA (M13mp18) was 100 nM dissolved in buffer (Tris/Tris-HCl 20 mM, Mg^2+^ 12.5 mM (pH 7.4)). Scale bar  = 100 nm. **Figure S3: Size distribution of the formed GUVs.** To confirm the size distribution of the GUVs, we prepared GUVs with the inner buffer of 40 µM Lucifer yellow (SIGMA, Japan), 300 mM mannitol, 0.1 mM CaCl_2_, 0.1 mM MgCl_2_. (A) Representative image of Lucifer yellow contained GUVs. Scale bar  = 50 µm. The fluorescent microscopic images of GUVs obtained immediately after water-in-oil emulsion transfer method. (B) The binarized image from (A). The size distribution was calculated from the images by using Image J software (NIH). (C) Size distribution of the Lucifer yellow contained GUVs diameter (n = 471). The average diameter is 37±13 µm, as mean ± standard deviation. **Figure S4:** **Representative “Pearl-chain” form of GUVs and HeLa cells suspension.** Each vesicle contained fluorescent beads of a particular size, e.g., 200 nm, 500 nm, 1 µm, and 2 µm in diameter. The coupled micrographs of phase contrast and fluorescence image show the same position of the sample immediately before the electrofusion. Scale bar  = 50 µm. **Figure S5:** **Confocal microscopic images of HeLa cells containing the introduced fluorescent beads.** (A) Microscopic images showing the cross-section of the treated HeLa cells. These images show HeLa cells into which (from the top) no beads, or beads of 0.2 µm, 0.5 µm, 1 µm, or 2 µm diameter (green) had been introduced. Z-stack acquisitions were performed to detect the position of the beads from the dorsal (left column) to the ventral (right column) cross-section of the cell. Cells were stained with cell tracker and Hoechst to reveal the cytoplasm (red) and nucleus (blue). Scale bar  = 10 µm. (B) 3-D reconstruction of images of HeLa cells containing 1-µm microbeads, obtained from confocal microscopic image stacks of a birds-eye view (upper left), side view (lower left), and top view (right). Sky blue, white line, and green dotted line indicate the bottom of the cells, slice position of the top view, and side view, respectively. **Table S1: Sequences for the plate-like DNA origami structure.**
(ZIP)
