# Supplementary material for: Introducing Micrometer-Sized Artificial Objects into Live Cells: A Method for Cell–Giant Unilamellar Vesicle Electrofusion
Source: PLoS One. 2014 Sep 17;9(9):e106853. doi: 10.1371/journal.pone.0106853 (PMC4167692; doi:10.1371/journal.pone.0106853)
Supplement: File S1 — Figure S1: Schematic diagram of the square DNA origami structure. M13mp18 ssDNA, its complementary ssDNA sets (called staples), and green fluorescent (FITC)-conjugated oligonucleotides (5′-GCAATGAGTAGATCCTGGCACTCTCGATGCGACAG-3′ and 5′-TGCCAGGATCTACTCATTGC-3′) were purchased from Operon Technologies (Japan) and Takara Bio (Japan), respectively. These DNAs were mixed (M13:staples:FITC = 4 nM:20 nM:60 nM) and annealed in a buffer (50 mM NaCl, 10 mM Tris-HCl, 10 mM MgCl2, 1 mM DTT, pH 7.9, 25°C) for 3.5 h across a temperature range from 95 to 25°C at a rate of -1°C/3 min. Figure S2: Identification of the DNA origami structure. (A) Electrophoresis analysis. Left, middle, and right lanes contain the marker, bare plate-like DNA origami structure, and fluorescently-tagged DNA origami, respectively. The samples were analyzed by 1% agarose gel electrophoresis (100V, 1 hour). The DNA origami structure, with or without fluorescent (FITC) tag, was electrophoresed in a 1% agarose gel that was exposed to DC 100 V for 1 h. FITC fluorescence was detected using a ChemiDoc MP system (BioRad, Japan). A band showing FITC-tagged origami was clearly observed under blue light. (B) AFM images for the DNA origami. The AFM image was obtained on an AFM system (Nano Live Vision, RIBM, Tsukuba, Japan) using a silicon nitride cantilever (resonant frequency = 1.5 MHz, spring constant = 0.1 Nm-1, EBDTip radius = 24 nm, Olympus BL-AC10DS-A2). The sample (2 µL) was adsorbed onto a freshly cleaved mica plate for 5 min at room temperature, and then washed twice with the same buffer solution. Scanning was performed in the same buffer solution using a tapping mode. The final concentration of the DNA (M13mp18) was 100 nM dissolved in buffer (Tris/Tris-HCl 20 mM, Mg2+ 12.5 mM (pH 7.4)). Scale bar = 100 nm. Figure S3: Size distribution of the formed GUVs. To confirm the size distribution of the GUVs, we prepared GUVs with the inner buffer of 40 µM Lucifer yellow (SIGMA, Japan), 300 mM mannitol, 0.1 mM CaCl2 [file pone.0106853.s001.zip › Table_S1.rtf]

Table S1. Sequences for the plate-like DNA origami structure.

Plate 1
Well    Sequence 5' to 3'
A1	AACAGATAAAATCGGAATACCCAATGTCACAATTTTTTTTTTTTTTTCTGTCGCATCGAGAG
B1	ATGACCCTGTAATACTTTTGCGGGAGCAATAATTTTTTTTTTTTTTTCTGTCGCATCGAGAG
C1	AAACGATTTTTTGTTTAACGTCAAGAGATAACTTTTTTTTTTTTTTTCTGTCGCATCGAGAG
D1	AAATCAACAGTTGAAAGGAATTGACAGCGAAATTTTTTTTTTTTTTTCTGTCGCATCGAGAG
E1	CATTTCGCTAGATTTAGTTTGACCGTACGGTGTTTTTTTTTTTTTTTCTGTCGCATCGAGAG
F1	TGCCCCCTCCTCAAGAGAAGGATTGATATAAGTTTTTTTTTTTTTTTCTGTCGCATCGAGAG
G1	TTTGTCGTAGCCCAATAGGAACCCCACCCTCATTTTTTTTTTTTTTTCTGTCGCATCGAGAG
H1	GAACGCGACGTAGGAATCATTACCCGGGTATTTTTTTTTTTTTTTTTCTGTCGCATCGAGAG
A2	ATGTGTAGACAGTCAAATCACCATTTAATGCCTTTTTTTTTTTTTTTCTGTCGCATCGAGAG
B2	CGTTTTAATTGCATCAAAAAGATTGGAATCGTTTTTTTTTTTTTTTTCTGTCGCATCGAGAG
C2	AGAGTAATATGAACGGTGTACAGATTTGTATCTTTTTTTTTTTTTTTCTGTCGCATCGAGAG
D2	GGTCGCTGATTGTATCGGTTTATCGAAAATCTTTTTTTTTTTTTTTTCTGTCGCATCGAGAG
E2	CCCCTTATTAGCGTCAGACTGTAGAATCACCATTTTTTTTTTTTTTTCTGTCGCATCGAGAG
F2	TAACACTGATAGTTAGCGTAACGATTTGCTAATTTTTTTTTTTTTTTCTGTCGCATCGAGAG
G2	GTAGCACCGTCACCGACTTGAGCCATATGGTTTTTTTTTTTTTTTTTCTGTCGCATCGAGAG
H2	GGAGAGGGAGGTCATTGCCTGAGAAATCGTAATTTTTTTTTTTTTTTCTGTCGCATCGAGAG
A3	AACATCGCACAAAGCCTGTAGCATGTGAGAATTTTTTTTTTTTTTTTCTGTCGCATCGAGAG
B3	ACAACCATGAATTTCTTAAACAGCAAAGGAATTTTTTTTTTTTTTTTCTGTCGCATCGAGAG
C3	ATCTAAAATATCTTTAGGAGCACTAAAGGCCGTTTTTTTTTTTTTTTCTGTCGCATCGAGAG
D3	AGACACCACCAAAGACAAAAGGGCAAGGTGAATTTTTTTTTTTTTTTCTGTCGCATCGAGAG
E3	ACCTCGCATCCAAGTTCCAGTAAGATTATTCTTTTTTTTTTTTTTTTCTGTCGCATCGAGAG
F3	ACCAAGTTGCAGAGGCGAATTATTTCATTTGATTTTTTTTTTTTTTTCTGTCGCATCGAGAG
G3	TAAAACAGATTGCGTAGATTTTCACTTTGAATTTTTTTTTTTTTTTTCTGTCGCATCGAGAG
H3	ATTGAGAATAACAACGCCAACATGCCAGACGATTTTTTTTTTTTTTTCTGTCGCATCGAGAG
A4	TAAATTGGTAAGAACTGGCTCATTAAAGATTCTTTTTTTTTTTTTTTCTGTCGCATCGAGAG
B4	TAGCAAACAAGTAAGCAGATAGCCAATAGCAATTTTTTTTTTTTTTTCTGTCGCATCGAGAG
C4	AGGCGTTAAACACCGGAATCATAAAGGGCTTATTTTTTTTTTTTTTTCTGTCGCATCGAGAG
D4	AAAGCGGATTCGAGCTTCAAAGCGGTGAGCGATTTTTTTTTTTTTTTCTGTCGCATCGAGAG
E4	CTGAGACTGCCTATTTCGGAACCTCGTCATACTTTTTTTTTTTTTTTCTGTCGCATCGAGAG
F4	ACAGGGAAGCGCATTAGACTACCAGGTTGGGGTTTTTTTTTTTTTTTCTGTCGCATCGAGAG
G4	CAAAATCATAGAATCCTTGAAAACATATATGTTTTTTTTTTTTTTTTCTGTCGCATCGAGAG
H4	TAGCAAGCTAGAAGGCTTATCCGGTTGCTATTTTTTTTTTTTTTTTTCTGTCGCATCGAGAG
A5	TCATCATATCAGATGATGGCAATTTTTGCACGTTTTTTTTTTTTTTTCTGTCGCATCGAGAG
B5	ATTACCTTAACAGTACATAAATCAATAGCGATTTTTTTTTTTTTTTTCTGTCGCATCGAGAG
C5	GTCAGATGTACCATATCAAAATTACATCAATATTTTTTTTTTTTTTTCTGTCGCATCGAGAG
D5	CGACAATATCAGCTAATGCAGAACAACCAATCTTTTTTTTTTTTTTTCTGTCGCATCGAGAG
E5	ATGGCTTTATGGAAAGCGCAGTCTAGAACCACTTTTTTTTTTTTTTTCTGTCGCATCGAGAG
F5	ACGGAACACCTCGTTTACCAGACGCCAAAATATTTTTTTTTTTTTTTCTGTCGCATCGAGAG
G5	AATAATCGCTTATCATTCCAAGAAGCGCCCAATTTTTTTTTTTTTTTCTGTCGCATCGAGAG
H5	ATTCATGTAACAAAGAGCCAGCAACGCGTTTTTTTTTTTTTTTTTTTCTGTCGCATCGAGAG
A6	AAGCCAGATGATGATACAGGAGTGACAGTTAATTTTTTTTTTTTTTTCTGTCGCATCGAGAG
B6	GTCACCAACGGAAATTATTCATTAGACATTCATTTTTTTTTTTTTTTCTGTCGCATCGAGAG
C6	TTTTTCATCGGAACGAGGGTAGCACAGTTGGCTTTTTTTTTTTTTTTCTGTCGCATCGAGAG
D6	AATATTGATGAAACCATCGATGCTATATAATATTTTTTTTTTTTTTTCTGTCGCATCGAGAG
E6	GAGTGAATATTACATTTAACAATTCATTTCAATTTTTTTTTTTTTTTCTGTCGCATCGAGAG
F6	TTATTTCAACGCAAGGATAAAAATCCTGAGTATTTTTTTTTTTTTTTCTGTCGCATCGAGAG
G6	ACAACTTTATAATTTTTTCACGTTAGCTTGCTTTTTTTTTTTTTTTTCTGTCGCATCGAGAG
H6	CGGATAAGACCCTCATTTTTCAGTCTCATCAGTTTTTTTTTTTTTTTCTGTCGCATCGAGAG
A7	CAAGGCAAAGCATAAAGCTAAATCAAAACATTTTTTTTTTTTTTTTTCTGTCGCATCGAGAG
B7	CTTGATATTAACGGGGTCAAAAAAACGATGTGTTTTTTTTTTTTTTTCTGTCGCATCGAGAG
C7	AGCCAGCTCCGTCGGATTCTCCGTAAATATCGTTTTTTTTTTTTTTTCTGTCGCATCGAGAG
D7	TCAGAATAAATAAGAGCCGCCACCCTGAATTTTTTTTTTTTTTTTTTCTGTCGCATCGAGAG
E7	GCAGAGGCGCCAACGCTCAACAGTTTACTAGATTTTTTTTTTTTTTTCTGTCGCATCGAGAG
F7	GAAACGCTTTAAGACATGAAAGTACGTACTCATTTTTTTTTTTTTTTCTGTCGCATCGAGAG
G7	TTATCACCATTACCATTAGCAAGGGAATCAAGTTTTTTTTTTTTTTTCTGTCGCATCGAGAG
H7	AGAATTAATTACCGAAGCCTAAATTCCAACTTTTTTTTTTTTTTTTTCTGTCGCATCGAGAG
A8	GCGAGAGGTTCAGAAAACGAGAATCAAAAATCTTTTTTTTTTTTTTTCTGTCGCATCGAGAG
B8	CCTCAGAACCGCCGCCAGCATTGACCTCATTATTTTTTTTTTTTTTTCTGTCGCATCGAGAG
C8	TACCAGCGCGGAATAAGTTTATTTAAGAACTGTTTTTTTTTTTTTTTCTGTCGCATCGAGAG
D8	ACCGATTGTGGCAACATATAAAAGCAGTATGTTTTTTTTTTTTTTTTCTGTCGCATCGAGAG
E8	GACAGCATGAGGAAGTTTCCATTACTCATCTTTTTTTTTTTTTTTTTCTGTCGCATCGAGAG
F8	AAGGAATTAGATTTAGGAATACCATTACCTTATTTTTTTTTTTTTTTCTGTCGCATCGAGAG
G8	GGGTAATTGAGCAAGAAACAATGAGAACAAAGTTTTTTTTTTTTTTTCTGTCGCATCGAGAG
H8	TGCGATTTGCTTGAGATGGTTTAACTTCATCATTTTTTTTTTTTTTTCTGTCGCATCGAGAG
A9	CATAAAGGAGGGAGGGAAGATTCCAGTTGGTATTTTTTTTTTTTTTTCTGTCGCATCGAGAG
B9	CTACTAATCGAGCTGAAAAGGTGGATTAGATATTTTTTTTTTTTTTTCTGTCGCATCGAGAG
C9	AGAAAGAAAAATAAAGGAACAACTTTGATACCTTTTTTTTTTTTTTTCTGTCGCATCGAGAG
D9	TTCGAGGTCGCCCACGCATAACCGATACATTTTTTTTTTTTTTTTTTCTGTCGCATCGAGAG
E9	ATCGCCTGAGCGATTATACCAAGCCTAAAGACTTTTTTTTTTTTTTTCTGTCGCATCGAGAG
F9	AGCCTTTAAGGCTTGCAGGGAGTTAACAACTATTTTTTTTTTTTTTTCTGTCGCATCGAGAG
G9	GAGGACAGCTTGACAAGAACCGGAACGAGTAGTTTTTTTTTTTTTTTCTGTCGCATCGAGAG
H9	TCACTTTCGCTGTATAGAAAATTCATTTGGGATTTTTTTTTTTTTTTCTGTCGCATCGAGAG
A10	CTGCTCCAAGAATACACTAAAACAAACGGGTATTTTTTTTTTTTTTTCTGTCGCATCGAGAG
B10	CAAAATCAGTAATCAGTAGCGACACCGGAAACTTTTTTTTTTTTTTTCTGTCGCATCGAGAG
C10	TTTAAGAAGTAGAAAATACGCTATGTTTAATATTTTTTTTTTTTTTTCTGTCGCATCGAGAG
D10	TTTGCCTTTAGCGTTTGCCATCTTGCCGCCACTTTTTTTTTTTTTTTCTGTCGCATCGAGAG
E10	TATTTTGTTCATTTTTTAACCAATCTTCCTGTTTTTTTTTTTTTTTTCTGTCGCATCGAGAG
F10	GGCCGGAGGTAAAGATTCAAAAGGAGAAGCCTTTTTTTTTTTTTTTTCTGTCGCATCGAGAG
G10	ATAATCAGTGTCAATCATATGTACATCTACAATTTTTTTTTTTTTTTCTGTCGCATCGAGAG
H10	CCACAATTAGCTACAAGAATTGAGGCAATAATTTTTTTTTTTTTTTTCTGTCGCATCGAGAG
A11	TGACCCCCATAAATTGTGTCGAAACTTTGAAATTTTTTTTTTTTTTTCTGTCGCATCGAGAG
B11	AAAAGCCTGAAATACCGACCGTGTAAAACTTTTTTTTTTTTTTTTTTCTGTCGCATCGAGAG
C11	CATATATTTCGCCCGGCATTTTCGCACCACCCTTTTTTTTTTTTTTTCTGTCGCATCGAGAG
D11	ACCACCGGGTCAGACGATTTAAATGACCAGGCTTTTTTTTTTTTTTTCTGTCGCATCGAGAG
E11	CAAAATAAACAGCCATATTATTTATCCCAATCCAACGCTATTTTTTTTTTTTTTTCTGTCGCATCGAGAG
F11	TGCCAGGATCTACTCATTGCTCCTCTTTTGAGGAACAAGTTTTCTTGT
G11	GCAATGAGTAGATCCTGGCACTCTCGATGCGACAG

Plate 2
Well    Sequence 5' to 3'
A1	TAAGAGGACTCTATGCAGATACATACTGGATAGCGGGCGACAGCTTCC
B1	ACCAGTGAAGCTGATTGCCCTTCAGTGGTTCCGAAATCGGTATAAATC
C1	GAGGGGACCAAAGCGCCATTCGCCCGCAACTGTTGGGAAGGTAACGCC
D1	GCTCACAAATTGCGTTGCGCTCACCCAGTCGGGAAACCTGTTCTTTTC
E1	TGTGCTGCCATGCCTGCAGGTCGATCCCCGGGTACCGAGCAGCCGGAA
F1	TTGGTGTAGATGGGCGCATCGTAAGCCAGTTT
G1	GTAATCATGCCAGTGCCAAGCTTGAAGGCGATTAAGTTGGGGCGATCG
H1	ACGGCGGAATAATTCGCGTCTGGCAGGAACGCCATCAAAAAAATATTT
A2	AAATTGTAGGAAGATTGTATAAGCAGAATCGATGAACGGTGTCTGGAG
B2	TGGCTGACTTTCAACTTTAAGCCTTGTAAATCATTGTGAACATTCAAC
C2	TATTCAACTATTTTAAATGCAATGTTTTAGAACCCTCATA
D2	CAAACAAGCGTTCTAGCTGATAAACAATATGA
E2	TTGACCGTAATGGGATTCAGGAAG
F2	GCTGCATTGAGCTAACTCACATTATTCCACACAACATACGTCGAATTC
G2	GTGCGGGCGGTGCCGGAAACCAGGGACGACAGTATCGGCCAGGTCACG
H2	AAGGCTGGTCCACTACAACGGAGACCAGGCGCATAGGAGACGCGGGGC
A3	AGGGTTTTACGTTGTAAAACGACGGGTCATAGCTGTTTCCTGTTATCC
B3	AAAAGAATAAAGGGCGAAAAACCGAATGAAAAATCTAAAGCATCACCT
C3	ATCGCACTTTCCGGCACCGCTTCTCTCTTCGCTATTACGCAAGGGGGA
D3	TTCCAGTTGTCCACTATTAAAGAACTCAAATATCAAACCCTCAATCAA
E3	TATCTGGTACGGCTACAGACAAGATGGAAGGCTTTGAGGAGCGAAACA
F3	TGCTGAACCGTGGACTCCAACGTCAGCCCGAGATAGGGTTAAAATCCT
G3	GCGTATTGGAGTTGCAGCAAGCGGTTTGCCCCAGCAGGCGGAGTGTTG
H3	GCATAAAGGGGGTGCCTAATGAGTAATGAATCGGCCAACGGGCGGTTT
A4	AATACTGCAAGAGGAAGCCCAGCTCCAGCCGAAAGACTTCGGGAACAA
B4	GTTTGATGCCGCCTGGCCCTGAGAGGCGCCAGGGTGGTTTTCGTGCCA
C4	CAAGAAAACAAAATTAAACCTTGCTTCTGTAA
D4	TCGGGAGAAACAATAAGCAAAAGAAGATGATG
E4	CGGGAGGTTTTGAAGCCTTTCCAGAGCCTAAT
F4	CTAAATTTAATGGTTTGTTTAGTATCATATGC
G4	TTCTTACCAGTATAAAATTTTCGAGCCAGTAA
H4	TAATATCCCATCCTAATACCGCACTCATCGAG
A5	ATTTTGCGGAACAAAGATTGTTTGGATTATAC
B5	TAAAGTACCGACAAAAAATAGATAAGTCCTGA
C5	CGTTATTAATTTTAAAAGTTTGAG
D5	TGGAAGGGTTAGAACCAATATACAGTAACAGT
E5	TTAATTAATTTTCCCTTAGGTCTGAGAGACTA
F5	CTCCGGCTTAGGTTGGTATTTTAGTTAATTTC
G5	CCGTTTTTATTTTCATGGCGTTTTAGCGAACC
H5	ATAAGTTTTCACAAACAAATAAATCAGGAGGT
A6	AGGCTATCTAGCTATTTTTGAGAGGTGAGAAATTTTTTTTTTTTTTTCTGTCGCATCGAGAG
B6	TTCGACAACTCGTATTAAATCCTTCGGAATTATTTTTTTTTTTTTTTCTGTCGCATCGAGAG
C6	TAGCTATCCTGAACACCCTGAACAACATAAAATTTTTTTTTTTTTTTCTGTCGCATCGAGAG
D6	GGATAGCACTTTCCAGACGCGCAGCGAGGTTATTTTTTTTTTTTTTTCTGTCGCATCGAGAG
E6	GCATGATTAGGAAACCGAGGAAACTTAAGCCCTTTTTTTTTTTTTTTCTGTCGCATCGAGAG
F6	TCATAAGGCAACGTAACAAAGCTGGAATAAGGTTTTTTTTTTTTTTTCTGTCGCATCGAGAG
G6	TTACCTGACGGATTCGCCTGATTGGGTTTAACTTTTTTTTTTTTTTTCTGTCGCATCGAGAG
H6	ACATGTTTTTTCATTCCATATAACCAATTCTGTTTTTTTTTTTTTTTCTGTCGCATCGAGAG
A7	CAGAACCGGGAATAGGTGTATCACTTAAGAGGTTTTTTTTTTTTTTTCTGTCGCATCGAGAG
B7	CTTTTGCGAATGCCACTACGAAGGAAACGAAATTTTTTTTTTTTTTTCTGTCGCATCGAGAG
C7	AGGGGGTATTCATTGAATCCCCCTTCAGAAGCTTTTTTTTTTTTTTTCTGTCGCATCGAGAG
D7	AAATACGTGGATCGTCACCCTCAGGGAAGGTTTTTTTTTTTTTTTTTCTGTCGCATCGAGAG
E7	ATTGCTCCAGAGCTTAATTGCTGAGTAGCTCATTTTTTTTTTTTTTTCTGTCGCATCGAGAG
F7	TATAGCCCCCACCCTCAGAACCGCATGTACCGTTTTTTTTTTTTTTTCTGTCGCATCGAGAG
G7	GTAAATGAAAGGCTCCAAAACCTACACCAAGGTTTTTTTTTTTTTTTCTGTCGCATCGAGAG
H7	GAGGATTTAGAAGTATTAGACTTTCAATGACATTTTTTTTTTTTTTTCTGTCGCATCGAGAG
A8	TTATCAACGGTAAAGTAATTCTGTTAATTTAGTTTTTTTTTTTTTTTCTGTCGCATCGAGAG
B8	GTAACAACTTCATCAACATTAAATTTTTTGTTTTTTTTTTTTTTTTTCTGTCGCATCGAGAG
C8	ATCATAACACATTATTACAGGTAGATACCAGTTTTTTTTTTTTTTTTCTGTCGCATCGAGAG
D8	ATAGATTAGAGCCGTCAATAGATAATATATTCTTTTTTTTTTTTTTTCTGTCGCATCGAGAG
E8	CCTTGAGTTGCTCAGTACCATAAACGTTAAGGTTTTTTTTTTTTTTTCTGTCGCATCGAGAG
F8	TTGCACCCTTATCCTGAATCTTACCAAATAAGTTTTTTTTTTTTTTTCTGTCGCATCGAGAG
G8	CTTGCCCTTGGGAAGAAAAATCTAACGAACTATTTTTTTTTTTTTTTCTGTCGCATCGAGAG
H8	ATAGCAGCCTTTACAGAGAGAATAAAGTCAGATTTTTTTTTTTTTTTCTGTCGCATCGAGAG
A9	ACCCAAATGAACCGAACTGACCAATCCGCGACTTTTTTTTTTTTTTTCTGTCGCATCGAGAG
B9	CATAAATAATAGTAAAATGTTTAGAACGCCAATTTTTTTTTTTTTTTCTGTCGCATCGAGAG
C9	TGCGAATACAACAGTTTCAGCGGATCCACAGATTTTTTTTTTTTTTTCTGTCGCATCGAGAG
D9	TTAAACAGCTTTTGCAAAAGAAGTGCAACACTTTTTTTTTTTTTTTTCTGTCGCATCGAGAG
E9	GAGCCACCTGCCGTCGAGAGGGTTAGGATTAGTTTTTTTTTTTTTTTCTGTCGCATCGAGAG
F9	GATGATTATTCCTAGTTGCGCCGAACAAACAATTTTTTTTTTTTTTTCTGTCGCATCGAGAG
G9	CCAAAAAAATTTTCTGTATGGGATTCTAAAGTTTTTTTTTTTTTTTTCTGTCGCATCGAGAG
H9	TTCAAATAGTTATATAACTATATGGAATTTATTTTTTTTTTTTTTTTCTGTCGCATCGAGAG
A10	AGCCTCAGAGAATTAGCAAAATTACATCAATTTTTTTTTTTTTTTTTCTGTCGCATCGAGAG
B10	AAATCAGCTAAAATTCGCATTAAACCCGGTTGTTTTTTTTTTTTTTTCTGTCGCATCGAGAG
C10	AGGTCTTTAACTCCAACAGGTCAGTACCTTTATTTTTTTTTTTTTTTCTGTCGCATCGAGAG
D10	GATGGCTTTTTTGATAAGAGGTCAAACCAGACTTTTTTTTTTTTTTTCTGTCGCATCGAGAG
E10	GGAATGGATTTTAGGTTTAGTACCCAAACTACTTTTTTTTTTTTTTTCTGTCGCATCGAGAG
F10	AGCTTAGAGAGAAGAGTCAATAGTTAAATGCTTTTTTTTTTTTTTTTCTGTCGCATCGAGAG
G10	ATCAGTTGACGAGGCATAGTAAGATTTGCCAGTTTTTTTTTTTTTTTCTGTCGCATCGAGAG
H10	AAACCAAGTTTACGAGCATGTAGAGCGCCTGTTTTTTTTTTTTTTTTCTGTCGCATCGAGAG
A11	TTACCAGAAAGACTCCTTATTACGAAACGCAATTTTTTTTTTTTTTTCTGTCGCATCGAGAG
B11	CGAACGAGAAATGGTCAATAACCTATTTTCATTTTTTTTTTTTTTTTCTGTCGCATCGAGAG
C11	GAGGCAAATGTTACTTAGCCGGAAACGGTCAATTTTTTTTTTTTTTTCTGTCGCATCGAGAG
D11	TAATCCTGAAACCACCAGAAGGAGTGCCCGAATTTTTTTTTTTTTTTCTGTCGCATCGAGAG
E11	TCTGGAAGTAAATATGCAACTAAATTTTTGCGTTTTTTTTTTTTTTTCTGTCGCATCGAGAG
F11	ACGAGCGTCTTAAATCAAGATTAGTATTCTAATTTTTTTTTTTTTTTCTGTCGCATCGAGAG
G11	TTGGGGCGAGTAGTAGCATTAACACATACAGGTTTTTTTTTTTTTTTCTGTCGCATCGAGAG
H11	CACCAGAGCCGCCACCCTCAGAGCGTCATAGCTTTTTTTTTTTTTTTCTGTCGCATCGAGAG
A12	CGGGGTTTAACAGTGCCCGTATAATACTGGTATTTTTTTTTTTTTTTCTGTCGCATCGAGAG
B12	AACTAGCAAAAAGCCCCAAAAACAAACGTTAATTTTTTTTTTTTTTTCTGTCGCATCGAGAG
C12	CGGAAGCAACCCTGACTATTATAGCAAATGCTTTTTTTTTTTTTTTTCTGTCGCATCGAGAG
D12	CAGCCCTCAGTTTCGTCACCAGTAGCCACCCTTTTTTTTTTTTTTTTCTGTCGCATCGAGAG
E12	CAGGACGTGACGAGAAACACCAGATATTCATTTTTTTTTTTTTTTTTCTGTCGCATCGAGAG
F12	GCAGCACCCCGGAACCAGAGAGAGGATTAGCCTTTTTTTTTTTTTTTCTGTCGCATCGAGAG
G12	GATGCAAAAGACAAAGAACGCGAGGATAAATATTTTTTTTTTTTTTTCTGTCGCATCGAGAG
H12	AATAATAAGAGCGCTAATATCAGAAAATGAAATTTTTTTTTTTTTTTCTGTCGCATCGAGAG
