# Supplementary figures and images for: Introducing Micrometer-Sized Artificial Objects into Live Cells: A Method for Cell–Giant Unilamellar Vesicle Electrofusion
Source: PLoS One. 2014 Sep 17;9(9):e106853. doi: 10.1371/journal.pone.0106853 (PMC4167692; doi:10.1371/journal.pone.0106853)

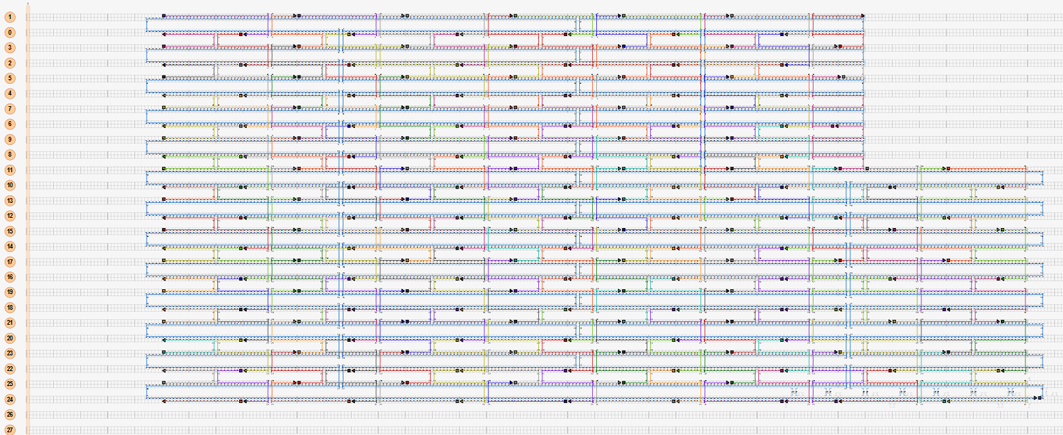

Supplement: File S1 — Figure S1: Schematic diagram of the square DNA origami structure. M13mp18 ssDNA, its complementary ssDNA sets (called staples), and green fluorescent (FITC)-conjugated oligonucleotides (5′-GCAATGAGTAGATCCTGGCACTCTCGATGCGACAG-3′ and 5′-TGCCAGGATCTACTCATTGC-3′) were purchased from Operon Technologies (Japan) and Takara Bio (Japan), respectively. These DNAs were mixed (M13:staples:FITC = 4 nM:20 nM:60 nM) and annealed in a buffer (50 mM NaCl, 10 mM Tris-HCl, 10 mM MgCl2, 1 mM DTT, pH 7.9, 25°C) for 3.5 h across a temperature range from 95 to 25°C at a rate of -1°C/3 min. Figure S2: Identification of the DNA origami structure. (A) Electrophoresis analysis. Left, middle, and right lanes contain the marker, bare plate-like DNA origami structure, and fluorescently-tagged DNA origami, respectively. The samples were analyzed by 1% agarose gel electrophoresis (100V, 1 hour). The DNA origami structure, with or without fluorescent (FITC) tag, was electrophoresed in a 1% agarose gel that was exposed to DC 100 V for 1 h. FITC fluorescence was detected using a ChemiDoc MP system (BioRad, Japan). A band showing FITC-tagged origami was clearly observed under blue light. (B) AFM images for the DNA origami. The AFM image was obtained on an AFM system (Nano Live Vision, RIBM, Tsukuba, Japan) using a silicon nitride cantilever (resonant frequency = 1.5 MHz, spring constant = 0.1 Nm-1, EBDTip radius = 24 nm, Olympus BL-AC10DS-A2). The sample (2 µL) was adsorbed onto a freshly cleaved mica plate for 5 min at room temperature, and then washed twice with the same buffer solution. Scanning was performed in the same buffer solution using a tapping mode. The final concentration of the DNA (M13mp18) was 100 nM dissolved in buffer (Tris/Tris-HCl 20 mM, Mg2+ 12.5 mM (pH 7.4)). Scale bar = 100 nm. Figure S3: Size distribution of the formed GUVs. To confirm the size distribution of the GUVs, we prepared GUVs with the inner buffer of 40 µM Lucifer yellow (SIGMA, Japan), 300 mM mannitol, 0.1 mM CaCl2 [file pone.0106853.s001.zip › Figure_S1.tif]

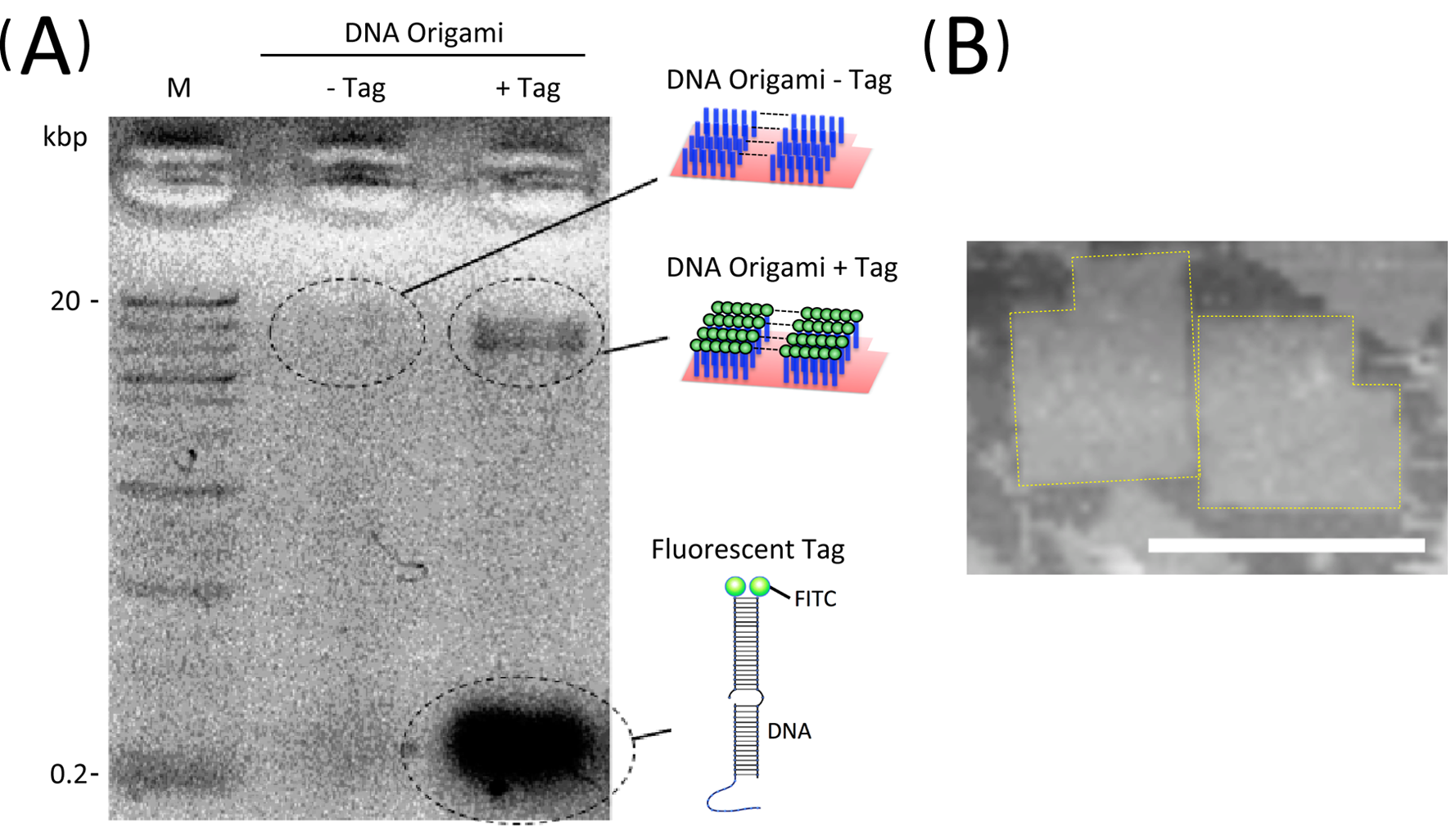

Supplement: File S1 — Figure S1: Schematic diagram of the square DNA origami structure. M13mp18 ssDNA, its complementary ssDNA sets (called staples), and green fluorescent (FITC)-conjugated oligonucleotides (5′-GCAATGAGTAGATCCTGGCACTCTCGATGCGACAG-3′ and 5′-TGCCAGGATCTACTCATTGC-3′) were purchased from Operon Technologies (Japan) and Takara Bio (Japan), respectively. These DNAs were mixed (M13:staples:FITC = 4 nM:20 nM:60 nM) and annealed in a buffer (50 mM NaCl, 10 mM Tris-HCl, 10 mM MgCl2, 1 mM DTT, pH 7.9, 25°C) for 3.5 h across a temperature range from 95 to 25°C at a rate of -1°C/3 min. Figure S2: Identification of the DNA origami structure. (A) Electrophoresis analysis. Left, middle, and right lanes contain the marker, bare plate-like DNA origami structure, and fluorescently-tagged DNA origami, respectively. The samples were analyzed by 1% agarose gel electrophoresis (100V, 1 hour). The DNA origami structure, with or without fluorescent (FITC) tag, was electrophoresed in a 1% agarose gel that was exposed to DC 100 V for 1 h. FITC fluorescence was detected using a ChemiDoc MP system (BioRad, Japan). A band showing FITC-tagged origami was clearly observed under blue light. (B) AFM images for the DNA origami. The AFM image was obtained on an AFM system (Nano Live Vision, RIBM, Tsukuba, Japan) using a silicon nitride cantilever (resonant frequency = 1.5 MHz, spring constant = 0.1 Nm-1, EBDTip radius = 24 nm, Olympus BL-AC10DS-A2). The sample (2 µL) was adsorbed onto a freshly cleaved mica plate for 5 min at room temperature, and then washed twice with the same buffer solution. Scanning was performed in the same buffer solution using a tapping mode. The final concentration of the DNA (M13mp18) was 100 nM dissolved in buffer (Tris/Tris-HCl 20 mM, Mg2+ 12.5 mM (pH 7.4)). Scale bar = 100 nm. Figure S3: Size distribution of the formed GUVs. To confirm the size distribution of the GUVs, we prepared GUVs with the inner buffer of 40 µM Lucifer yellow (SIGMA, Japan), 300 mM mannitol, 0.1 mM CaCl2 [file pone.0106853.s001.zip › Figure_S2.tif]

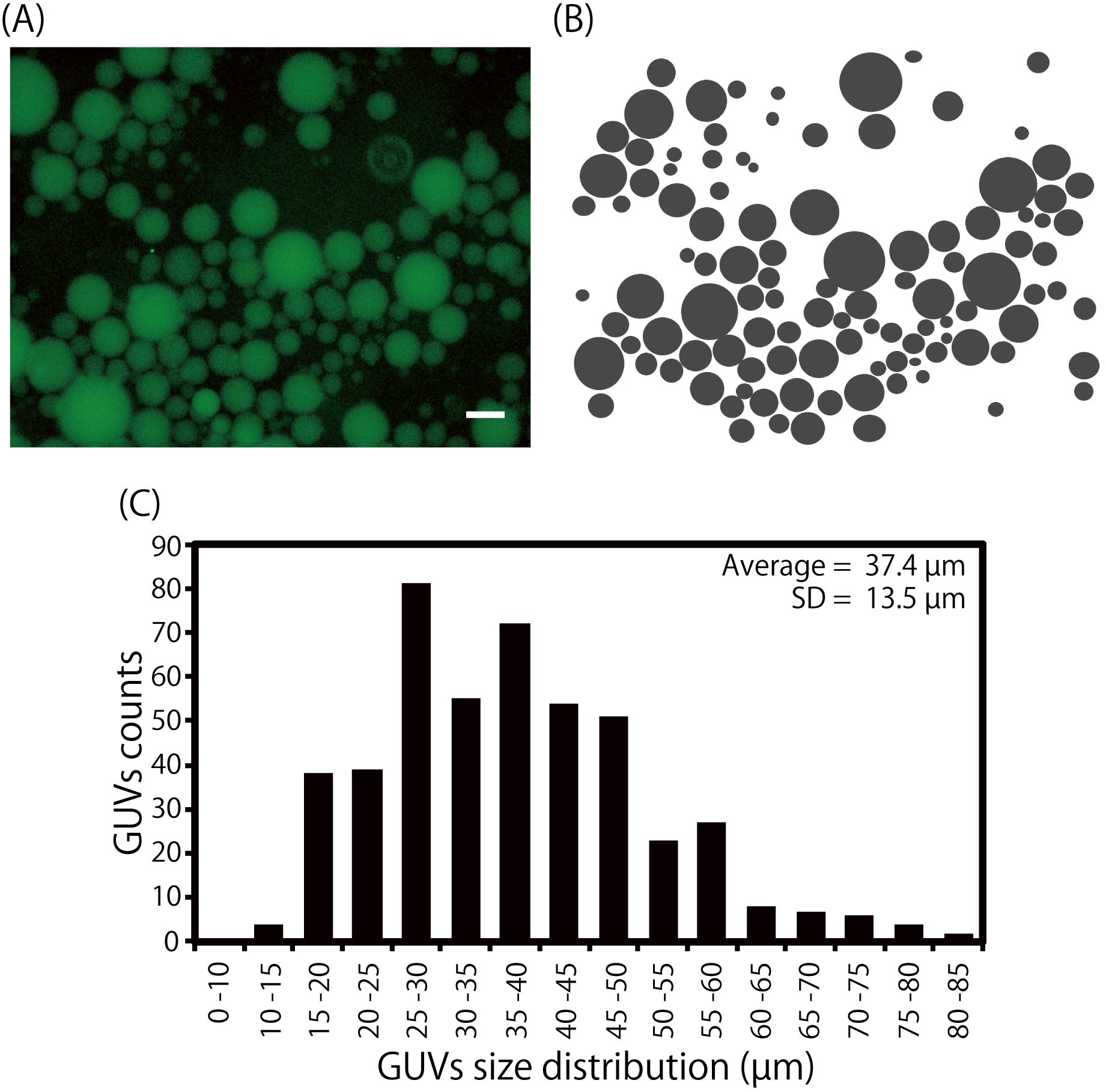

Supplement: File S1 — Figure S1: Schematic diagram of the square DNA origami structure. M13mp18 ssDNA, its complementary ssDNA sets (called staples), and green fluorescent (FITC)-conjugated oligonucleotides (5′-GCAATGAGTAGATCCTGGCACTCTCGATGCGACAG-3′ and 5′-TGCCAGGATCTACTCATTGC-3′) were purchased from Operon Technologies (Japan) and Takara Bio (Japan), respectively. These DNAs were mixed (M13:staples:FITC = 4 nM:20 nM:60 nM) and annealed in a buffer (50 mM NaCl, 10 mM Tris-HCl, 10 mM MgCl2, 1 mM DTT, pH 7.9, 25°C) for 3.5 h across a temperature range from 95 to 25°C at a rate of -1°C/3 min. Figure S2: Identification of the DNA origami structure. (A) Electrophoresis analysis. Left, middle, and right lanes contain the marker, bare plate-like DNA origami structure, and fluorescently-tagged DNA origami, respectively. The samples were analyzed by 1% agarose gel electrophoresis (100V, 1 hour). The DNA origami structure, with or without fluorescent (FITC) tag, was electrophoresed in a 1% agarose gel that was exposed to DC 100 V for 1 h. FITC fluorescence was detected using a ChemiDoc MP system (BioRad, Japan). A band showing FITC-tagged origami was clearly observed under blue light. (B) AFM images for the DNA origami. The AFM image was obtained on an AFM system (Nano Live Vision, RIBM, Tsukuba, Japan) using a silicon nitride cantilever (resonant frequency = 1.5 MHz, spring constant = 0.1 Nm-1, EBDTip radius = 24 nm, Olympus BL-AC10DS-A2). The sample (2 µL) was adsorbed onto a freshly cleaved mica plate for 5 min at room temperature, and then washed twice with the same buffer solution. Scanning was performed in the same buffer solution using a tapping mode. The final concentration of the DNA (M13mp18) was 100 nM dissolved in buffer (Tris/Tris-HCl 20 mM, Mg2+ 12.5 mM (pH 7.4)). Scale bar = 100 nm. Figure S3: Size distribution of the formed GUVs. To confirm the size distribution of the GUVs, we prepared GUVs with the inner buffer of 40 µM Lucifer yellow (SIGMA, Japan), 300 mM mannitol, 0.1 mM CaCl2 [file pone.0106853.s001.zip › Figure_S3.tif]

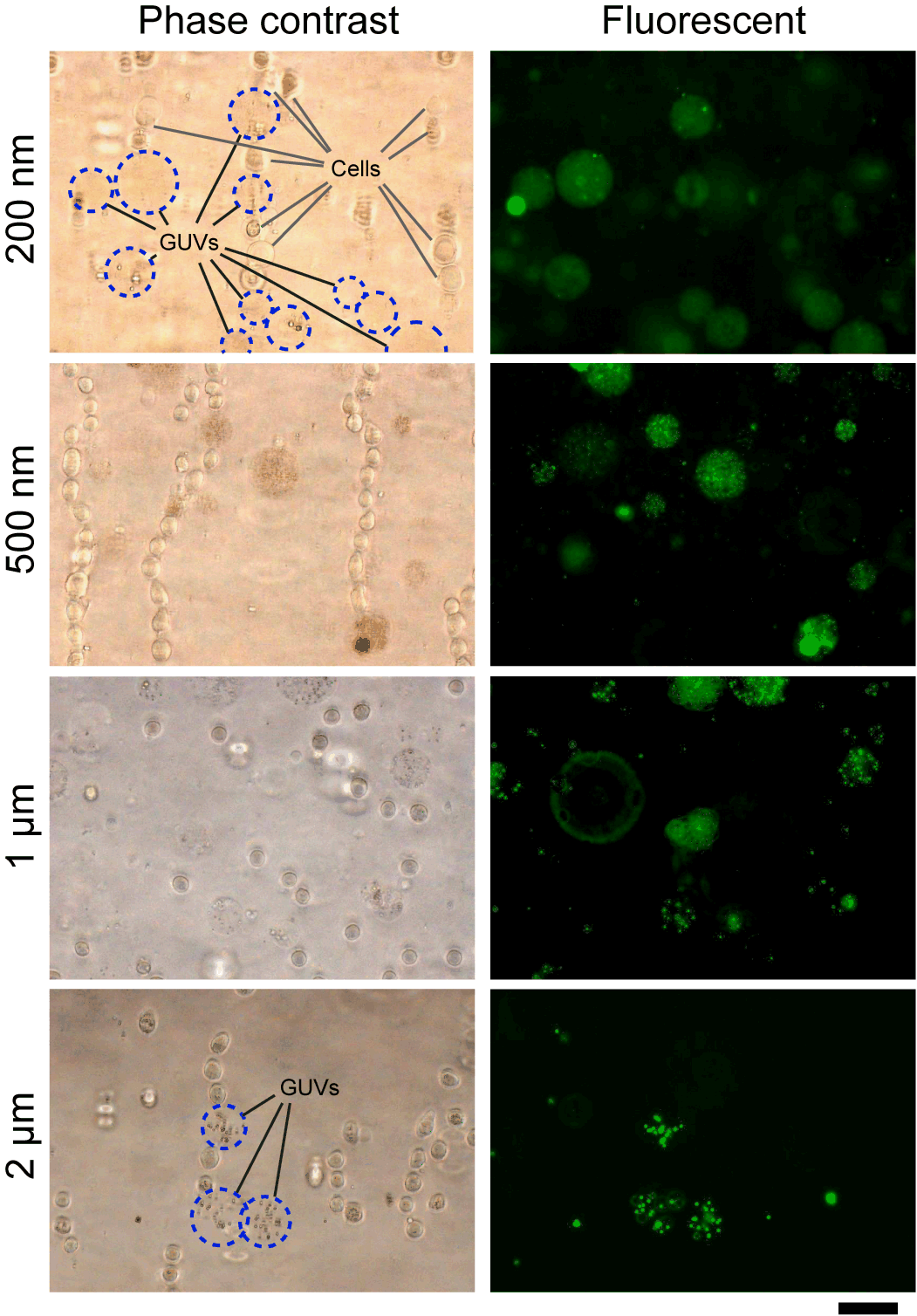

Supplement: File S1 — Figure S1: Schematic diagram of the square DNA origami structure. M13mp18 ssDNA, its complementary ssDNA sets (called staples), and green fluorescent (FITC)-conjugated oligonucleotides (5′-GCAATGAGTAGATCCTGGCACTCTCGATGCGACAG-3′ and 5′-TGCCAGGATCTACTCATTGC-3′) were purchased from Operon Technologies (Japan) and Takara Bio (Japan), respectively. These DNAs were mixed (M13:staples:FITC = 4 nM:20 nM:60 nM) and annealed in a buffer (50 mM NaCl, 10 mM Tris-HCl, 10 mM MgCl2, 1 mM DTT, pH 7.9, 25°C) for 3.5 h across a temperature range from 95 to 25°C at a rate of -1°C/3 min. Figure S2: Identification of the DNA origami structure. (A) Electrophoresis analysis. Left, middle, and right lanes contain the marker, bare plate-like DNA origami structure, and fluorescently-tagged DNA origami, respectively. The samples were analyzed by 1% agarose gel electrophoresis (100V, 1 hour). The DNA origami structure, with or without fluorescent (FITC) tag, was electrophoresed in a 1% agarose gel that was exposed to DC 100 V for 1 h. FITC fluorescence was detected using a ChemiDoc MP system (BioRad, Japan). A band showing FITC-tagged origami was clearly observed under blue light. (B) AFM images for the DNA origami. The AFM image was obtained on an AFM system (Nano Live Vision, RIBM, Tsukuba, Japan) using a silicon nitride cantilever (resonant frequency = 1.5 MHz, spring constant = 0.1 Nm-1, EBDTip radius = 24 nm, Olympus BL-AC10DS-A2). The sample (2 µL) was adsorbed onto a freshly cleaved mica plate for 5 min at room temperature, and then washed twice with the same buffer solution. Scanning was performed in the same buffer solution using a tapping mode. The final concentration of the DNA (M13mp18) was 100 nM dissolved in buffer (Tris/Tris-HCl 20 mM, Mg2+ 12.5 mM (pH 7.4)). Scale bar = 100 nm. Figure S3: Size distribution of the formed GUVs. To confirm the size distribution of the GUVs, we prepared GUVs with the inner buffer of 40 µM Lucifer yellow (SIGMA, Japan), 300 mM mannitol, 0.1 mM CaCl2 [file pone.0106853.s001.zip › Figure_S4.tif]

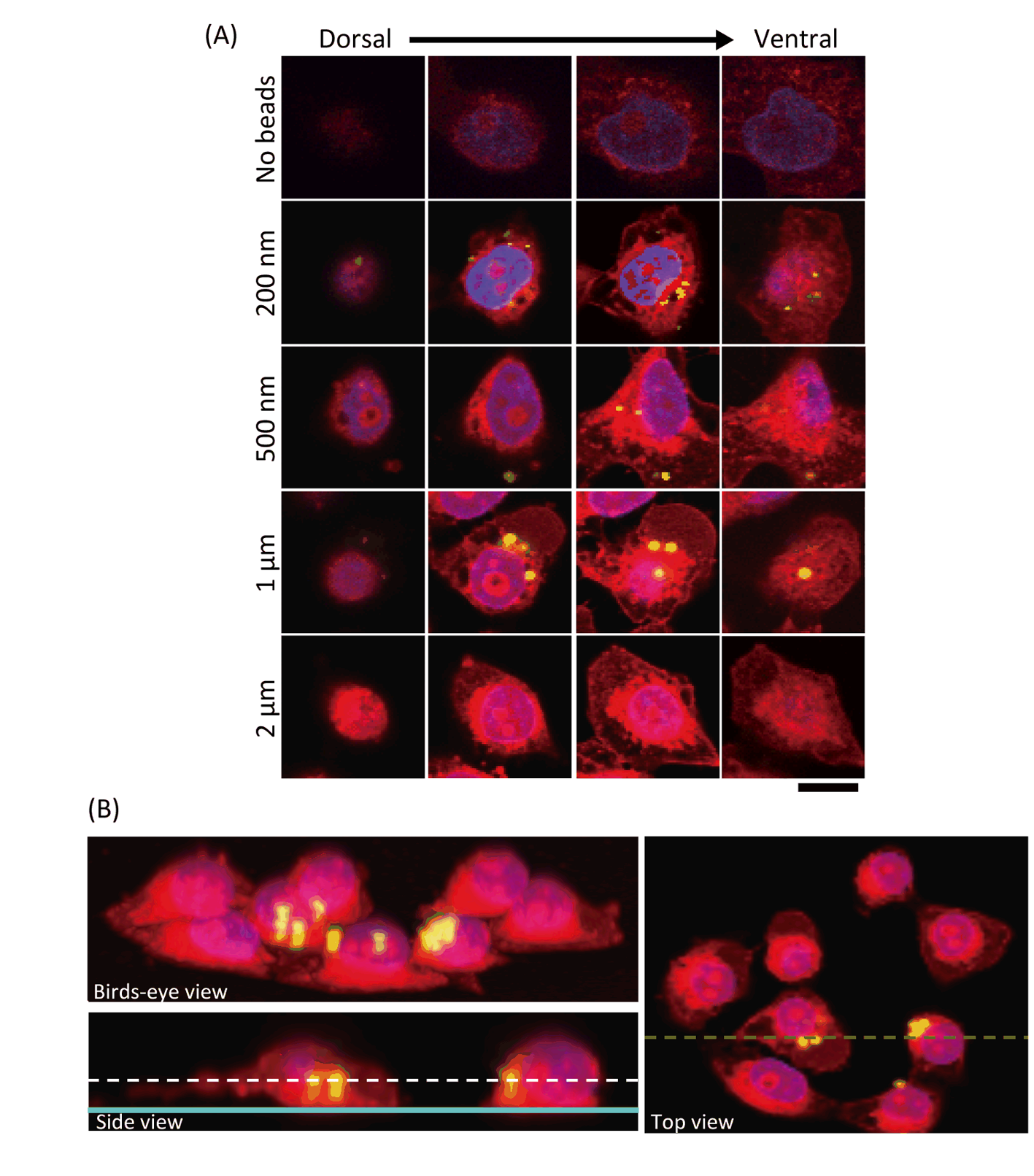

Supplement: File S1 — Figure S1: Schematic diagram of the square DNA origami structure. M13mp18 ssDNA, its complementary ssDNA sets (called staples), and green fluorescent (FITC)-conjugated oligonucleotides (5′-GCAATGAGTAGATCCTGGCACTCTCGATGCGACAG-3′ and 5′-TGCCAGGATCTACTCATTGC-3′) were purchased from Operon Technologies (Japan) and Takara Bio (Japan), respectively. These DNAs were mixed (M13:staples:FITC = 4 nM:20 nM:60 nM) and annealed in a buffer (50 mM NaCl, 10 mM Tris-HCl, 10 mM MgCl2, 1 mM DTT, pH 7.9, 25°C) for 3.5 h across a temperature range from 95 to 25°C at a rate of -1°C/3 min. Figure S2: Identification of the DNA origami structure. (A) Electrophoresis analysis. Left, middle, and right lanes contain the marker, bare plate-like DNA origami structure, and fluorescently-tagged DNA origami, respectively. The samples were analyzed by 1% agarose gel electrophoresis (100V, 1 hour). The DNA origami structure, with or without fluorescent (FITC) tag, was electrophoresed in a 1% agarose gel that was exposed to DC 100 V for 1 h. FITC fluorescence was detected using a ChemiDoc MP system (BioRad, Japan). A band showing FITC-tagged origami was clearly observed under blue light. (B) AFM images for the DNA origami. The AFM image was obtained on an AFM system (Nano Live Vision, RIBM, Tsukuba, Japan) using a silicon nitride cantilever (resonant frequency = 1.5 MHz, spring constant = 0.1 Nm-1, EBDTip radius = 24 nm, Olympus BL-AC10DS-A2). The sample (2 µL) was adsorbed onto a freshly cleaved mica plate for 5 min at room temperature, and then washed twice with the same buffer solution. Scanning was performed in the same buffer solution using a tapping mode. The final concentration of the DNA (M13mp18) was 100 nM dissolved in buffer (Tris/Tris-HCl 20 mM, Mg2+ 12.5 mM (pH 7.4)). Scale bar = 100 nm. Figure S3: Size distribution of the formed GUVs. To confirm the size distribution of the GUVs, we prepared GUVs with the inner buffer of 40 µM Lucifer yellow (SIGMA, Japan), 300 mM mannitol, 0.1 mM CaCl2 [file pone.0106853.s001.zip › Figure_S5.tif]
